# Estimating hepatitis A immunity and outbreak risk among MSM in New South Wales, Australia, 2017–2018

**DOI:** 10.1017/S0950268826101940

**Published:** 2026-07-07

**Authors:** Neil Franklin, Kirsty Hope, Keira Glasgow, Kathryn Glass, Martyn Kirk

**Affiliations:** 1 Department of Applied Epidemiology, National Centre for Epidemiology and Population Health, Australia; 2 Health Protection NSW, Population and Public Health Division, NSW Ministry of Health, St Leonards, Australia

**Keywords:** Australia, hepatitis A, men who have sex with men, outbreaks, public health practice, vaccination

## Abstract

In 2017–2018, New South Wales (NSW), Australia, experienced a hepatitis A virus (HAV) outbreak predominantly affecting men who have sex with men (MSM). We aimed to estimate immunity and identify immunity gaps among MSM in NSW. We triangulated four data sources. We tested 409 residual sera collected from males undergoing syphilis testing between August and October 2017 for anti-HAV IgG. We surveyed all publicly funded sexual health clinics about HAV testing and vaccine provision for the preceding financial year. We conducted a convenience behavioural survey of 50 men at a sex-on-premises venue in October 2017, and we analysed community survey data from 2018 to 2019 (*n* = 5,924). Descriptive statistics and logistic regression ere used to examine associations with immunity and vaccination. Overall serological immunity was 62.8% (257/409) and increased with age, from 50.0% in those aged under 26 years to 79.0% in those over 45 years. Clinic immunity and community survey estimates were higher (74.5% and 76.8%). Immunity gaps were identified among bisexual men, those less connected to the gay community, and people born outside Australia. NSW Health implemented targeted vaccination and community engagement in response. Multisource assessment provided actionable evidence to guide targeted vaccination and outbreak response.

## Impact statement

This study assessed hepatitis A immunity among men who have sex with men in New South Wales during a 2017–2018 outbreak, to identify immunity gaps and inform outbreak control. We triangulated four data sources: serological testing of 409 residual sera collected from males undergoing syphilis testing between August and October 2017, a survey of all 17 publicly funded sexual health clinics, a behavioural survey of 50 men at a sex-on-premises venue, and community survey data from 2018 to 2019 (*n* = 5,924). Overall serological immunity was 62.8% (257/409) and increased significantly with age, from 50.0% in men aged under 26 years to 79.0% in those aged over 45 years (*p* for trend <0.001). Clinic-based immunity estimates were higher (74.5%), and self-reported vaccination in community surveys was 74.3% in 2018 and 76.8% in 2019. Vaccination coverage was lower in bisexual than gay-identifying men (61.8% vs. 77.2%; *p* < 0.001). Immunity gaps were also identified among men less connected to the gay community and those born outside Australia. These findings informed targeted vaccination and community messaging, and demonstrated the value of multisource immunity assessment for outbreak response and prevention.

## Introduction

Hepatitis A virus (HAV) is commonly transmitted primarily through the faecal–oral route, often via contaminated food or water, or through close person-to-person contact. Infection is preventable through vaccination. While HAV incidence has declined in many high-income countries due to improved sanitation and childhood vaccination in some regions, susceptible populations remain at risk of outbreaks, particularly where immunity is low [1].

In recent years, multiple HAV outbreaks have been reported among men who have sex with men (MSM) across Europe [2–5] and the USA [6, 7], with molecular evidence linking many cases to the same internationally circulating genotype IA strains. These outbreaks demonstrate how HAV can rapidly spread through globally connected MSM networks and highlights the importance of understanding population-level immunity in these groups.

Between July 2017 to August 2018, New South Wales (NSW), Australia, experienced its first hepatitis A outbreak among MSM since 1996 [8]. This outbreak affected 44 people in NSW – full outbreak details are provided in Supplementary Material S1. Genomic sequencing of local cases revealed that the outbreak strains were genetically related to those involved in contemporaneous European outbreaks. Although hepatitis A vaccination is recommended for MSM under Australian immunization guidelines [9]; uptake is not routinely monitored, and population immunity levels remain poorly characterized. This limits the ability to anticipate outbreak risk and implement timely public health prevention strategies.

Mathematical modelling suggests that HAV immunity needs to exceed 70% in large MSM populations to prevent sustained transmission [10]. In response to the 2017–2018 outbreak, we undertook a rapid assessment to estimate immunity levels and identify potential immunity gaps. This study describes the integration of multiple data sources, including disease notifications, serological testing, clinic records, and behavioural surveys, to inform the public health response and guide future prevention efforts.

## Methods

To assess HAV immunity and understand vaccine uptake and provision among MSM in NSW, three complementary surveys were conducted.

### Serological survey

To estimate HAV immune status among MSM, six public and two private laboratories were each requested to select stored serum specimens collected between August and September 2017 from males aged 16 to 69 years who had undergone syphilis testing (regardless of result) at sexual health clinics (SHCs) or general practices frequently attended by MSM patients. The first 50 specimens meeting these criteria per laboratory were selected. This number was based on resource availability and to minimize operational burden on laboratories during the acute response period. These specimens were selected because syphilis testing is routinely recommended for sexually active MSM in NSW, whereas testing in non-MSM populations is typically based on clinical suspicion. Thus, syphilis testing was used as a proxy indicator for MSM status. Specimens were tested for anti-HAV IgG or total antibody to indicate immunity from either past infection or vaccination. De-identified data including age, sex, postcode, specimen collection date, and HAV test results were provided to NSW Health.

### SHC survey

To understand clinical practices related to HAV vaccination among MSM, we conducted an online survey of all 17 publicly funded SHCs in NSW, completed by clinic manager, medical or nursing staff. The survey consisted of structured multiple-choice and short-answer questions developed by the study team and collected information covering clinic practices for assessing HAV immunity, offering HAV vaccination, and associated costs. Clinics were also asked to report the proportion of MSM clients who received HAV IgG testing and HAV vaccination during the 2016/2017 financial year. Where possible, responses were based on data extracted from clinical record systems; otherwise, clinics provided informed estimates.

### Sex-on-premises venue survey

To assess awareness of the outbreak and vaccination coverage among at-risk individuals, a convenience sample of men attending a sex-on-premises venue (SOPV) was surveyed. This SOPV was one which several outbreak cases had attended during their exposure or infectious period. Data collection occurred in October 2017, on two separate days (a Friday and a Wednesday) between 12:30 pm and 2:30 pm. Clients entering the venue were invited to complete a short (5-min) questionnaire covering awareness of the HAV outbreak, vaccination history, healthcare-seeking behaviours, demographics, and level of connection with the gay community. Surveys were either self-completed or administered by an investigator. This survey was conducted as part of the public health response to a hepatitis A outbreak, and data were collected anonymously with voluntary participation.

## Health response

The results of each survey were analysed separately, but collated and compared to determine where vaccination coverage was lacking, with the aim to see what could be done to address any gaps identified. NSW Health also worked with community partners, including the AIDS Council of NSW (ACON) and Positive Life NSW to draft and deliver community messaging.

To monitor HAV vaccination coverage following the outbreak, a request was made to add a question about HAV vaccination status to the Sydney Gay Community Periodic Surveys (GCPS) from the 2018 survey onwards. The GCPS are annual, cross-sectional surveys of gay and homosexually active men, recruited from gay venues, community events, and online platforms across Sydney and NSW [11]. For this analysis, de-identified 2018–2019 Sydney GCPS data obtained from the Centre for Social Research in Health, University of NSW Sydney, following submission and approval of a data request for the variables including HAV vaccination status, age, country of birth, postcode, and sexual orientation. The 2020 GCPS HAV vaccination estimates were sourced directly from the published report [12].

## Data analysis

Descriptive statistics were used to summarize demographic and clinical characteristics across all survey components. Categorical variables were compared using chi-squared tests or Fisher’s exact tests where appropriate. Trends in immunity across age groups were assessed using the chi-squared test for linear trend.

For the SHC and SOPV surveys, analyses were limited to descriptive and bivariable comparisons given the small sample sizes and number of outcome events. Univariable logistic regression was used where appropriate to examine associations between participant characteristics and HAV immunity or vaccination status. For the serosurvey, we fitted a multivariable logistic regression model including age group and place of residence. Results are presented as odds ratios (ORs) with 95% confidence intervals (CIs). All analyses were conducted using Stata version 11.2, and a two-sided *p*-value <0.05 was considered statistically significant.

This investigation was conducted as part of the public health response authorized under section s106 of the NSW Public Health Act [13]. As each data collection involved low-risk, unidentifiable data, all activities were conducted under the auspice of the NSW Public Health Act and formal Human Research Ethics Committee (HREC) approval was not required.

## Results

### Serological survey

A total of 409 residual serum specimens collected between 21 August and 16 October 2017 from eight pathology laboratories were tested (50–55 specimens contributed per site). HAV antibodies were detected in 62.8% (257/409) of specimens, with immunity varying between laboratories from 44% to 85%. Immunity increased significantly with age, from 50.0% among those aged under 26 years to 79.0% among those aged over 45 years (χ^2^ trend = 13.33; *p* < 0.001). The majority of specimens (80.7%) were from men residing in a major city. These individuals had significantly higher HAV immunity compared to those from regional areas (65.8%, *χ* ^2^ = 8.09; *p* = 0.005; Table 1). After adjusting for place of residence, age remained the strongest predictor of immunity: individuals aged over 30 years had 2.48 times the odds of HAV immunity compared to those under 30 (95% CI: 1.62–3.78; *p* < 0.001).

**Table 1.** Summary of hepatitis A immunity and vaccination coverage among men who have sex with men (MSM) in NSW, based on data from serological testing, sexual health clinics, SOPV behavioural survey and the Sydney Gay Community Periodic Survey 2018–2019 (GCPS) Table 1. long description.

Immunity measure	Serosurvey 2017		SHC survey 2017		SOPV survey 2017		GCPS 2018–2019	
	Serological immune pos		Immune result + vaccine provision		Self-report (Vx history)		Self-report (Vx history)	
	Number	% immune	Number	% immune	Number	% Vx	Number	% Vx
Total	409 specimens	62.8%	6 clinics	74.50%	50 responses	66.7%	5,924 responses	75.6%
Age								
<26	53/103	50.0%	–		3/6	50.0%	783/1018	76.9%
26–45	155/244	63.9%	–		13/18	72.2%	2625/3359	78.1%
46+	49/62	79.0%	–		16/24	66.7%	1053/1522	69.2%
Residence								
Major city	217/330	65.8%	–		27/42	64.3%	4023/5292	76.0%
Regional area	31/66	47.0%	–		2/2	100.0%	370/500	74.0%
Country of birth								
Australia	–		–		19/24	79.2%	2849/3657	77.9%
Other	–		–		13/24	54.2%	1614/2241	77.2%
Proportion of gay friends								
<50%	–		–		20/29	69.0%	2468/3376	73.1%
50%+	–		–		12/19	63.2%	2009/2546	78.9%
% time spent with gay peers								
<50%	–		–		15/25	60.0%	2638/3614	73.0%
50%+	–		–		17/23	73.9%	1837/2305	79.7%
Sexual orientation								
Gay	–		–		25/34	73.5%	4067/5269	77.2%
Bisexual	–		–		7/13	53.8%	252/408	61.8%
GP aware of MSM activity								
Yes	–		–		28/38	73.7%	–	
No	–		–		4/9	44.4%	–	

### SHC survey

All 17 SHCs in NSW reported routinely assessing HAV immunity among MSM clients at their initial visit. However, there was variability in assessment methods: eight clinics (47%) used HAV IgG testing alone, while ten (59%) relied solely or partially on clients’ verbal report of previous HAV infection or vaccination. Seven clinics (41%) were able to extract data on the proportion of MSM clients tested for HAV IgG in 2016–2017, with testing proportions ranging from 59% to 95% (median: 75.5%). Among those tested, HAV immunity ranged from 35% to 75% (median: 48.4%).

Eight clinics (47%) reported first-dose HAV vaccine administration coverage between 4% and 33% (median: 13.5%), with a mean second-dose completion of 69% in those who had received the first dose. Higher vaccination coverage was observed in clinics with lower HAV immunity on screening. Six clinics provided both screening and vaccination data, with estimated immunity ranging from 54% to 83.3% (median: 74.5%). These estimates did not include verbal reports of immunity and are therefore likely conservative.

At the time of the survey, ten SHCs (59%) provided both doses of the HAV vaccine free of charge to eligible MSM clients. Four clinics (24%) provided only the first dose for free, while three clinics (18%) did not provide free vaccine but offered private prescriptions.

### SOPV survey

Fifty men completed a knowledge, risk, and vaccination behaviour survey at an SOPV over two days. Participants were skewed towards older ages compared with the outbreak cases (median age 36 years): 28% were aged 18–35 years, 20% were 36–45 years, and 52% were ≥ 46 years. Twenty-eight per cent identified as bisexual or preferred to not define their sexual orientation (26% and 2%, respectively). Taken together, 43% of these men reported being married to a woman. Most respondents (90%) were residents of metropolitan Sydney; 4% were from regional NSW and 6% from interstate. Australian-born men comprised 48% of participants, with the remainder born in 14 different countries.

Participants estimated that, on average, 47% of their social circle were gay and that they spent 45% of their time with gay men. Awareness of the HAV outbreak affecting MSM was moderate (42%), with participants reporting they heard about the outbreak via general practitioners (GPs) (29%), gay media (24%), friends or peers (24%), and social media (19%).

Awareness of hepatitis A and B vaccines was high (84%). Thirty-eight per cent of participants reported receiving two HAV vaccine doses, and a further 6% had received at least one dose. An additional 20% recalled receiving a hepatitis vaccine but could not specify whether it was for hepatitis A or B. Two participants (4%) reported immunity from past infection, while 32% reported no vaccination or were unsure of their vaccination status. Among vaccinated participants, the most common reasons were travel (52%) and sexual risk (52%), with 12% citing both. Eight per cent could not recall why the vaccine was recommended.

Men whose GP was aware of their MSM status had about 3.5 the odds of being vaccinated against HAV than those whose GP did not know (OR 3.5; 95% CI 0.78–15.69; *p* = 0.09), and those who were those born in Australia had 3.2 times the odds of being vaccinated for HAV than those born overseas (OR 3.2; 95% CI 0.90–11.46; *p* = 0.07), but these differences did not reach statistical significance. Coverage was lowest among participants aged under 26 years (50.0%) and highest among those aged 26–45 years (72.2%), with no evidence of an age association (*χ* ^2^ = 1.00, df = 2, *p* = 0.61). Residence, proportion of gay friends, and proportion of time spent in the gay community were not clearly associated with vaccination status. (Table 1).

### Health response and follow-up

Based on the serological and clinic data, at least two of the three assessments indicated HAV immunity levels potentially below the 70% threshold required to prevent sustained community transmission (Table 1). In response, NSW Health initiated targeted immunization activities for MSM in late 2017.

All public SHCs were supported to provide at least one free dose of HAV vaccine to all eligible MSM clients. GPs were notified of the outbreak and encouraged to offer the vaccine to at-risk patients. Public health messaging was co-developed with community partners, and promoted through gay media, social media, and posters in SOPVs.

The HAV vaccination coverage in gay and bisexual men in NSW as reported in the GCPS was 74.3% in 2018, rising to 76.8% in 2019 (*χ* ^2^ = 4.9; *p* = 0.027) and remaining stable at 76.1% in 2020. In the 2018 and 2019 surveys, most demographic groups reported immunity levels exceeding 70%; however, bisexual men had significantly lower self-reported vaccination coverage (61.8%) compared with gay-identifying men (77.2%; *χ* ^2^ = 49.5; *p* < 0.001; Table 1).

## Discussion

This multisource assessment of HAV immunity among MSM in NSW, Australia, was conducted during a period of increased transmission risk. Few studies have used real-time, multimethod data to assess population-level immunity and inform vaccination strategies. MSM communities in high-income countries are highly interconnected through travel and sexual networks [14]. Our findings highlight key immunity gaps and provide insights relevant to other high-income countries where local hepatitis A outbreaks among MSM have continued to occur despite the availability of safe and effective vaccines.

Although several MSM subgroups approached or exceeded the 70% herd immunity threshold [10], immunity remained insufficient in key populations. Notably, gaps were most evident among bisexual men, those with weaker social ties to the gay community, those living in regional areas and MSM born outside of Australia.

The serological survey provided a cross-sectional snapshot of immunity using stored sera from men tested for syphilis. The survey found an overall HAV immunity prevalence of 62.8%, with notable variation between laboratories (44–85%). As the serosurvey sampled men tested for syphilis at sexual health services, immunity estimates may be higher than in MSM with less healthcare engagement, but could also be diluted if some sampled men were not MSM. Nonetheless, the survey revealed clear age-related differences in immunity consistent with historical exposure and vaccine uptake patterns. Older men, who are more likely to have acquired immunity through past infection or travel vaccination, were significantly more likely to be immune than younger cohorts, underscoring the need to prioritize younger MSM in public health messaging and outreach.

Data from SHCs confirmed heterogeneity in immunity assessment practices and approaches to vaccine delivery. Despite universal screening protocols, methods varied widely, with some clinics relying on client recall rather than serological testing. Reported HAV immunity at first screen ranged from 35% to 75%, and vaccine administration also varied significantly. Lower immunity rates were reported from clinics in regional and outer-metropolitan areas. This finding of lower HAV immunity in regional areas was also reflected in the GCPS. While concerning, this may not be a critical public health issue since an MSM outbreak is unlikely to be sustained in regional areas with smaller concentrations of MSM. Encouragingly, clinics with lower baseline immunity showed higher vaccine uptake during the response, suggesting appropriate targeting, and SHCs that were not providing HAV vaccines for free to their MSM patients changed their practice to do so once informed of the outbreak.

The SOPV survey offered a complementary behavioural lens, identifying social and structural factors associated with lower vaccine uptake. Men whose GPs were aware of their sexual behaviour and those born in Australia had higher vaccination rates, though due to the size of the cohort, this finding lacked statistical significance. Although the SOPV survey was relatively small and may underrepresent younger or less sexually active MSM, these patterns align with findings from the pan-European EMIS-2017 study, which showed that MSM who were ‘out’ regarding their sexual identity had significantly higher odds of being vaccinated against hepatitis A and B, especially in countries where free, MSM-targeted vaccination policies existed [15]. This reinforces the importance of inclusive, accessible vaccine delivery and the role of social connectedness in vaccine uptake.

Following these findings, NSW Health initiated a coordinated public health response, including expanding free vaccine access at SHCs, alerting primary care providers, and implementing community-based health promotion in partnership with trusted MSM organizations. These efforts were followed by a measurable increase in self-reported vaccination coverage to 76.8% in 2019 from the previous year’s result in the GCPS, sustained in 2020. This coverage data from the GCPS were higher than any previously found in this study, and likely reflects higher rates of vaccination in that cohort, which is ‘primarily of Anglo-Australian background, live in metropolitan Sydney, are well-educated, gay-identified, and in full-time employment’. [16]. However, coverage among bisexual men remained significantly lower than among gay-identifying men, reinforcing the need for tailored strategies that engage subpopulations often underserved by mainstream MSM-focused interventions.

Our study illustrates the value of triangulating data from laboratory serosurveys, clinic-based audits, and community-level behavioural surveillance to capture a nuanced picture of immunity. This approach allowed us to rapidly identify coverage gaps and inform a targeted, community-based vaccine promotion campaign in partnership with sexual health services and sexuality- and gender-diverse community organizations. Such rapid, localized responses are directly applicable to other jurisdictions managing sporadic outbreaks or aiming to build MSM immunity between epidemic waves.

In Australia, national guidance recommends MSM are provided the HAV vaccine, however, there is no provision for funding. Despite this, NSW Health has continued to offer free vaccine to MSM through most SHCs. These findings underscore the value of proactive, sustained vaccination programmes over post-outbreak catch-up campaigns. In NSW, ongoing free HAV vaccination for MSM was associated with a limited outbreak of just 44 cases over 13 months; whereas in Victoria, an Australian city of similar population but where free vaccine provision had not been maintained, an outbreak grew to over 270 cases over 24 months [17]. A mass vaccination programme initiated in 2018 in Victoria to finally address this immunity gap likely contributed to the end of their outbreak [18]. Similarly, in Berlin between August 2016 and February 2018, only one-third of MSM were fully vaccinated pre-outbreak, resulting in 222 cases despite rapid interventions that increased vaccine uptake by 164% only after transmission peaked [19]. A coordinated framework, combining multisource surveillance, routine vaccine funding, inclusive clinical practice, and community engagement, offers a robust model for maintaining HAV immunity in MSM populations. Given the recurring nature of MSM-associated HAV outbreaks in Europe, adopting similar proactive strategies could significantly strengthen outbreak preparedness and prevention across high-income settings.

## Conclusion

After more than 20 years without a hepatitis A outbreak among MSM in Australia, waning population immunity allowed a moderate-sized outbreak in NSW in 2017–2018, seeded by ongoing outbreaks in Europe and North America. By triangulating serological, clinic-based, and community survey data, we were able to target vaccine promotion effectively and limit the 2017–2018 outbreak to just 44 cases. Our experience demonstrates that sustained, free vaccination programmes, combined with real-time immunity monitoring and community engagement, can both close immunity gaps and curb transmission. Public health authorities facing similar MSM-associated HAV risks should consider adopting this integrated framework to guide pre-emptive vaccination strategies and strengthen outbreak preparedness.

## Supporting information

10.1017/S0950268826101940.sm001 Franklin et al. supplementary material Franklin et al. supplementary material

## Data Availability

The data used in this study were obtained under the provisions of the NSW Public Health Act 2010. Due to privacy, confidentiality, and legislative restrictions, these data cannot be shared publicly.

## Supplementary material

The supplementary material for this article can be found at http://doi.org/10.1017/S0950268826101940.

## Long descriptions

### Table 1. Long description

Table 1 summarises hepatitis A immunity or vaccination coverage among MSM in NSW using four data sources: a 2017 serosurvey, a 2017 sexual health clinic survey, a 2017 SOPV survey, and the 2018–2019 Sydney Gay Community Periodic Survey. Overall estimates were 62.8%, 74.5%, 66.7% and 75.6%, respectively. In the serosurvey, immunity increased with age, from 50.0% among men under 26 years to 79.0% among men aged 46 years and over. Serological immunity was lower in regional areas than major cities. Self-reported vaccination was lower among bisexual men than gay men in both SOPV and GCPS data. In SOPV, coverage was also lower among men whose GP was not aware of their MSM activity. Dashes indicate data were not collected or not available.

Navigate back to Table 1..
