# Supplementary material for: Estimating hepatitis A immunity and outbreak risk among MSM in New South Wales, Australia, 2017–2018
Source: Epidemiol Infect. 2026 Jul 7;154:e100. doi: 10.1017/S0950268826101940 (PMC13433879; doi:10.1017/S0950268826101940)
Supplement: Supplementary file 1 [file S0950268826101940sup001.docx]

**Supplementary material S1**

**Hepatitis A outbreak investigation and control among men who have sex with men, New South Wales, Australia, July 2017–September 2018**

In July 2017–September 2018 New South Wales experienced a hepatitis A outbreak that predominantly affected men who have sex with men and was linked to internationally circulating genotype 1A strains. This supplement presents the key findings of the outbreak investigation—including case definitions, epidemiological findings, genotyping results and public‑health control measures—to complement the main article’s assessment of hepatitis A immunity and vaccination coverage.

**Outbreak investigation**

An outbreak case was defined as a person with hepatitis A virus infection meeting the national confirmed case definition, genotyped as 1A and closely matching (within 2 nucleotide changes) one of two identified European outbreak strains (VRD_521_2016 or RIVM-HAV16–09), with onset of symptoms after 1 July 2017 who spent at least some of their acquisition period (15 to 50 days prior to onset of illness) in Australia. A case was defined as probable if it met the national confirmed case definition and spent some of their acquisition period in Australia but genotyping was not available.

A standardised questionnaire administered by public health officers was used to collect the patients' demographic and clinical characteristics, as well as details of food exposures, sexual exposures, overseas travel, and contact with a symptomatic hepatitis case in the 50 days prior to onset of illness.

**Outbreak descriptive epidemiology**

From 1 July 2017 to 8 August 2018, a total of 130 HAV notifications were received. Of these, 44 met the confirmed outbreak case definition and no cases met the probable case definition. Confirmed cases included 43 males and 1 female, age range 21 to 69 years (median 36 years) with 18 of the 44 cases (41%) hospitalised for their illness. No deaths were reported. Thirty-seven (84%) cases resided in metropolitan Sydney and seven in regional areas. Five cases reported overseas travel during part of their incubation period. Twenty-eight of the 43 males (65%) reported MSM behaviour and five cases were thought to be secondary cases. Three cases reported injecting drug use (IDU).

**Laboratory investigation**

Serum samples for serologically diagnosed cases of HAV are routinely sent for further laboratory analysis to the Victorian Infectious Diseases Reference Laboratory. Samples were tested for HAV RNA by reverse transcription-polymerase chain reaction (RT-PCR) and if positive, HAV was sequenced employing the HAV network (HAVNET) protocol[1]. This procedure uses a nested RT-PCR to amplify a fragment of approximately 500 nucleotides spanning the HAV VP1/2A junction region which can be directly sequenced. After being analysed, the sequence data were uploaded to the National Centre for Biotechnology Information (NCBI) BLAST (Basic Local Alignment Search Tool) to determine the HAV genotype. Phylogenetic analysis compares isolate sequences against other sequences in the global HAVNET database. Three distinct genotype patterns were observed on sequencing. The first wave of infections, and the majority of cases (31 cases, 73%) differed by 2 of 463 nucleotides from the European outbreak strain VRD_521_2016. Later cases in the outbreak matched (10 cases, 23%) or were one nucleotide different (2 cases, 5%) to the European outbreak strain RIVM-HAV16-090 (Figure 1).

**Figure 1. Epicurve of HAV outbreak cases by sequence result and risk group, NSW, July 2017 – September 2018.**

Number of cases

| Month of case onset | MSM VRD | MSM RIVM | Travel VRD | Travel RIVM | MSM & Travel VRD | No risk VRD | No Risk RIVM |
| --- | --- | --- | --- | --- | --- | --- | --- |
| 2017-07-01 |  |  | 2 |  |  | 2 |  |
| 2017-08-01 | 7 |  |  |  |  | 5 |  |
| 2017-09-01 | 6 |  |  |  |  | 5 |  |
| 2017-10-01 | 3 |  |  |  | 1 |  |  |
| 2017-11-01 |  | 2 |  |  |  |  |  |
| 2017-12-01 | 1 |  |  |  |  |  | 1 |
| 2018-01-01 |  |  |  | 1 |  |  |  |
| 2018-04-01 |  | 2 |  | 1 |  |  | 1 |
| 2018-05-01 |  | 2 |  |  |  |  |  |
| 2018-07-01 |  | 1 |  |  |  |  |  |
| 2018-08-01 |  | 1 |  |  |  |  |  |

|  | MSM exposure |  | Sequence VRD_521-2016 |
| --- | --- | --- | --- |
|  |  |  |  |
|  | Travel exposure |  | Sequence RIVM-HAV16-090 |
|  |  |  |  |
|  | MSM & Travel exposure |  |  |
|  |  |  |  |
|  | No identified risk |  |  |

**Outbreak control**

Very few interpersonal links were discovered in the investigation of cases, indicating likely unknown transmission. Numerous cases also had overseas exposures, allowing for the possibility of multiple incursions in the local population.

Control measures included targeted HAV vaccine provision to sexual health clinics and public messaging via sexuality- and gender-diverse community organisations about the importance in ensuring HAV vaccination is up to date. Contact tracing and vaccination of household and sexual contacts were also implemented.

**Conclusion**

This outbreak was part of a global resurgence of HAV among MSM, with multiple strains introduced into NSW. Molecular typing was critical for distinguishing co-circulating strains and informing the public health response.

1.	National Institute for Public Health and Environment. HAVNET <https://www.rivm.nl/en/havnet> [updated 16-12-2019. Available from: <http://www.rivm.nl/en/Topics/H/HAVNET>.
